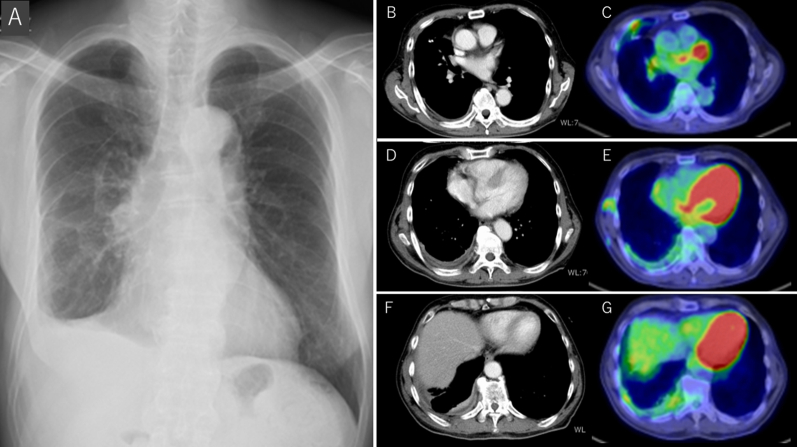# Salvage Pleurectomy/Decortication After Immunotherapy for Sarcomatoid Malignant Pleural Mesothelioma

**DOI:** 10.1016/j.atssr.2022.07.004

**Published:** 2022-08-05

**Authors:** Kenta Kajiyama, Akihiro Taira, Masaru Takenaka, Koji Kuroda, Midori Kusano, Aya Nawata, Fumihiro Tanaka

**Affiliations:** 1 Second Department of Surgery (Chest Surgery), University of Occupational and Environmental Health, Japan, Kitakyushu, Japan; 2 Department of Pathology and Oncology, School of Medicine, University of Occupational and Environmental Health, Japan, Kitakyushu, Japan

## Abstract

Sarcomatoid malignant pleural mesothelioma (MPM) is a highly aggressive malignant tumor. Surgery may not be recommended, and chemotherapy is less effective. More recently, immunotherapy has become a new standard treatment of care for advanced MPM across all histologic subtypes. This reports describes a case of salvage lung-sparing surgery (pleurectomy with decortication) after immunotherapy with nivolumab in combination with ipilimumab for sarcomatoid MPM. The surgical specimen showed that a major pathologic response was achieved with immunotherapy. The present case indicates not only the feasibility of pleurectomy with decortication after immunotherapy but also pathologic evidence of the efficacy of immunotherapy, which provides insight into the treatment of advanced MPM.

Malignant pleural mesothelioma (MPM) is a rare malignant tumor arising from mesothelial cells of the pleura. Radical surgery, extrapleural pneumonectomy, or pleurectomy with decortication (P/D) may be indicated for selected patients with early resectable disease, but whether this treatment improves prognosis remains unclear. The majority of patients present with unresectable disease, and they may be treated with systemic chemotherapy. However, standard chemotherapy using pemetrexed in combination with platinum provides only a modest survival benefit.^1^ Sarcomatoid MPM is a highly aggressive subtype associated with the poorest prognosis. Radical surgery may not be recommended and chemotherapy is less effective for patients with sarcomatoid MPM.^1^ Recently, immunotherapy using nivolumab (an anti–programmed cell death 1 antibody) in combination with ipilimumab (an anti–cytotoxic T-lymphocyte 4 antibody) has become a new standard treatment of care for advanced MPM.^2^ Treatment with nivolumab in combination with ipilimumab provides a superior survival benefit over chemotherapy across all histologic subtypes, including sarcomatoid MPM. Accordingly, even for patients with sarcomatoid MPM, radical surgery may be indicated when immunotherapy controls disseminated disease.

Here we present a case of salvage P/D after immunotherapy with nivolumab in combination with ipilimumab for sarcomatoid MPM. A surgical specimen showed that a major pathologic response was achieved with immunotherapy. This case not only suggests the feasibility of P/D after immunotherapy but also provides pathologic evidence of the efficacy of immunotherapy and may thus offer new insight into the treatment of advanced MPM, especially for intractable sarcomatoid MPM. Written informed consent was obtained from the patient, and the University of Occupational and Environmental Health, Japan (Kitakyushu, Japan) Institutional Review Board approved the study on June 18, 2014 (No. H26-35).

A 69-year-old Japanese man who reported having right-sided chest pain presented at a hospital. A chest roentogenogram and computed tomography revealed a right pleural effusion with pleural thickening (Figure 1). Thoracoscopic pleural biopsy provided pathologic evidence of sarcomatoid MPM, and the patient was referred to our hospital (University of Occupational and Environmental Health, Japan) for treatment.

**Figure 1:**
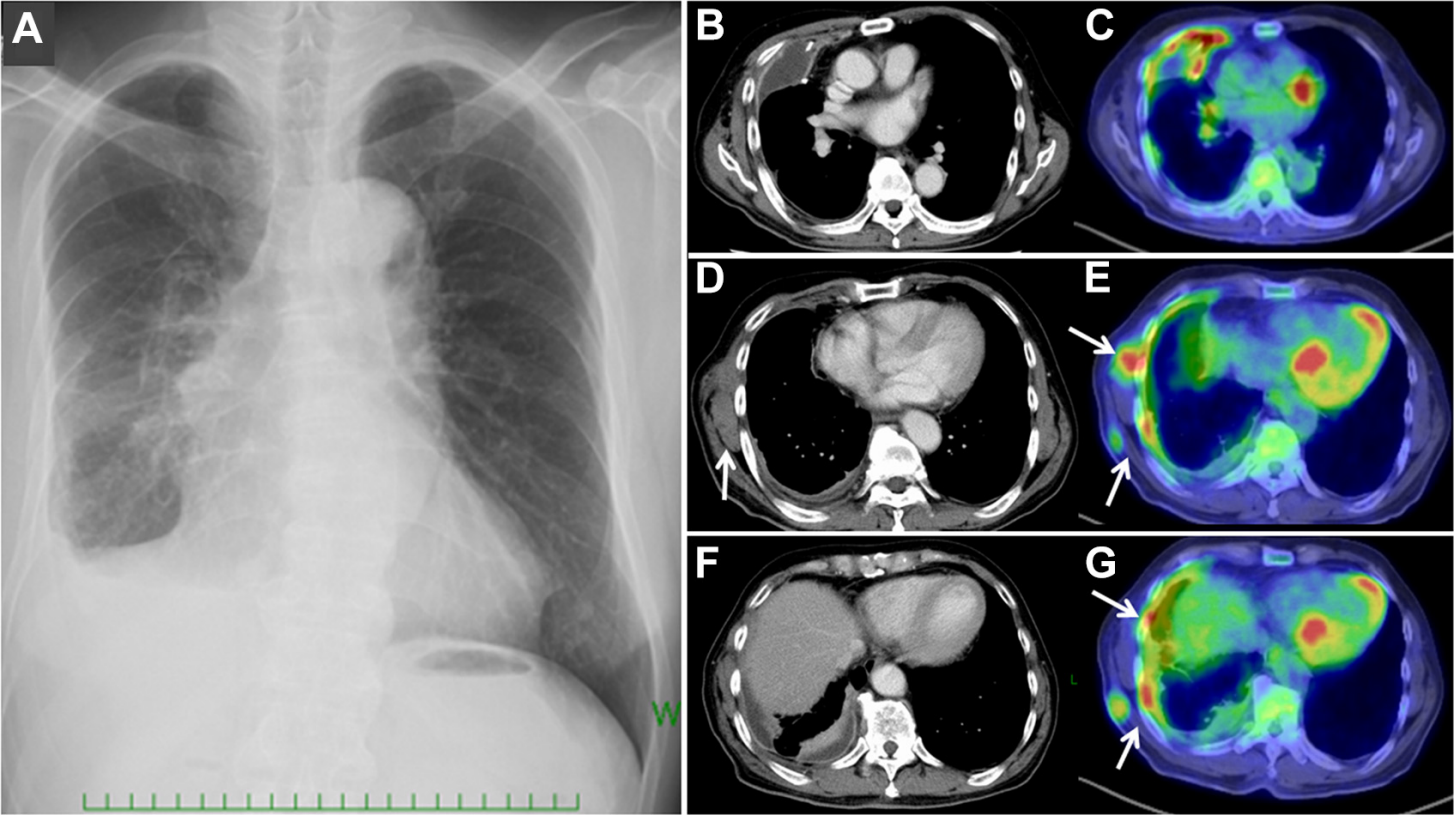
(A-G) Pretreatment imaging. (A) Chest roentogenogram, (B), (D), and (F), computed tomography, and (C), (E), and (G), positron emission tomography combined with computed tomography. Multifocal chest wall involvement was documented (arrows).

Because positron emission tomography with computed tomography revealed multifocal chest wall involvement, the patient was given a diagnosis of unresectable clinical stage IIIB disease (T4 N0 M0). A 3-month course of immunotherapy consisting of nivolumab (3 mg/kg intravenous infusion, once every 2 weeks) and ipilimumab (1 mg/kg intravenous infusion, once every 6 weeks) achieved a significant radiographic response (50% decrease in summed measurement from the baseline according to the modified Response Evaluation Criteria in Solid Tumors) (Supplemental Figure). No nodal or distant metastasis has developed, and salvage curative-intent surgery was performed.

Through a sixth posterolateral thoracotomy, extended P/D with combined resection of the pericardium, diaphragm, and chest wall was performed using a nonincisional technique,^3^ which achieved macroscopic complete resection (Figure 2, Video). Histologic sections showed achievement of a major pathologic response: less than 1% of the tumor cells were viable in the resected specimen (Figure 3). The postoperative course was uneventful, and the patient is alive without tumor recurrence at 5 months after surgery.

**Figure 2:**
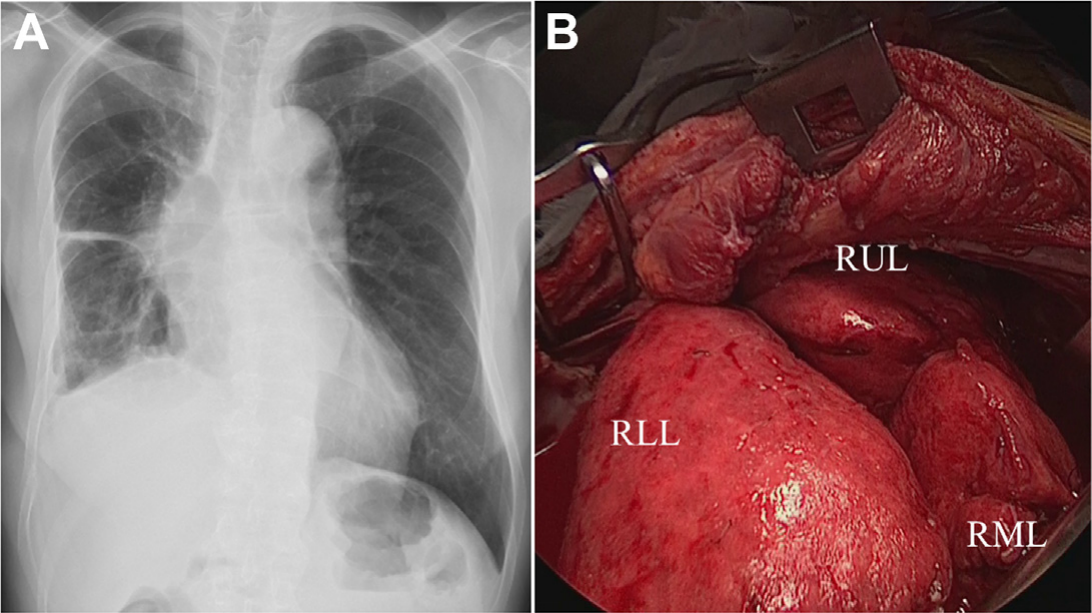
Macroscopic complete resection by sparing the entire right lung was achieved with extended pleurectomy and decortication. (A) Chest roentgenogram; (B) intraoperative photograph. (RLL, right lower lobe; RML, right middle lobe; RUL, right upper lobe.)

**Figure 3:**
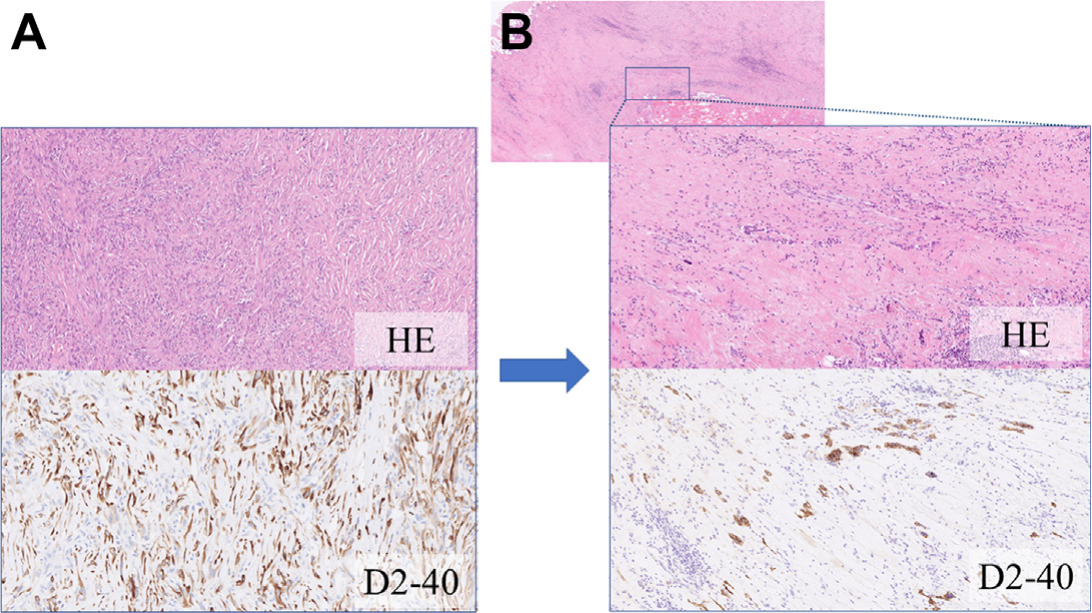
(A) The biopsy specimen showed diffuse proliferation of atypical spindle cells arranged in fascicles or in haphazard patterns with mitotic figures. (Hematoxylin and eosin; original magnification ×100.) The atypical spindle cells were diffusely positive for D2-40 and focally positive for AE1/AE3 and calretinin, whereas they were negative for thyroid transcription factor 1 and carcinoembryonic antigen. These features are compatible with sarcomatoid mesothelioma. (B) The surgical specimen of the pleura, diaphragm, and pericardium exhibited dense fibrosis, inflammation, and necrosis resulting from immunotherapy. Only small clusters of atypical spindle or polygonal cells with mitotic figures were seen in less than 1% of resected tissue. (Original magnification ×100.)

## Comment

Salvage surgery after immunotherapy may be a feasible treatment option for selected patients with initially unresectable non-small cell lung cancer.^4^ Because immunotherapy using nivolumab in combination with ipilimumab has been approved as a first-line systemic treatment for unresectable MPM, some patients may be candidates for salvage surgery after immunotherapy. In fact, Banks and coworkers^5^ reported a case of salvage extrapleural pneumonectomy after immunotherapy for refractory MPM. Here we presented a case of salvage P/D after immunotherapy. A lung-sparing surgical procedure such as P/D may be associated with lower morbidity and mortality, and it has been preferred for resectable MPM in recent years.^1^^,^^3^ As curative-intent salvage surgery after immunotherapy that is sometimes associated with fatal adverse events such as interstitial lung disease, P/D may provide a greater advantage over extrapleural pneumonectomy if macroscopic complete resection is achieved with P/D. The present case also revealed pathologic evidence of efficacy of immunotherapy because less than 1% of tumor cells were viable in the resected specimen. These results suggest that neoadjuvant immunotherapy before surgery for resectable MPM is a promising treatment strategy that should be examined in future clinical trials.

In conclusion, the present case may indicate that P/D is safe and feasible after immunotherapy and that immunotherapy may provide a dramatic pathologic response in patients with MPM. The present case also indicates the promise of P/D after neoadjuvant immunotherapy for resectable MPM.

## Figures and Tables

**Supplemental Figure 1:**