# Supplementary material for: Salvage Pleurectomy/Decortication After Immunotherapy for Sarcomatoid Malignant Pleural Mesothelioma
Source: Ann Thorac Surg Short Rep. 2022 Aug 5;1(1):121–3. doi: 10.1016/j.atssr.2022.07.004 (PMC11708474; doi:10.1016/j.atssr.2022.07.004)
Supplement: Supplemental Figure Legend [file mmc1.docx]

**Supplemental Figure Legend**

**Supplemental Figure 1.** Chest roentogenogram (**A**), computed tomography (CT; **B**, **D** and **F**) and positron emission tomography-CT (PET-CT; **C**, **E** and **G**) after immunotherapy.
